# Encapsulation of Bioactive Compounds from *Aloe Vera* Agrowastes in Electrospun Poly (Ethylene Oxide) Nanofibers

**DOI:** 10.3390/polym12061323

**Published:** 2020-06-10

**Authors:** Ignacio Solaberrieta, Alfonso Jiménez, Ilaria Cacciotti, Maria Carmen Garrigós

**Affiliations:** 1 Department of Analytical Chemistry, Nutrition & Food Sciences, University of Alicante, San Vicente del Raspeig, ES-03690 Alicante, Spain; solaberrieta@ua.es (I.S.); alfjimenez@ua.es (A.J.); 2 Department of Engineering, University of Rome “Niccolò Cusano”, INSTM RU, Via Don Carlo Gnocchi 3, 00166 Rome, Italy

**Keywords:** *Aloe Vera* agrowastes, poly(ethylene oxide), bioactive compounds, electrospun nanofibers, antioxidant activity, active food packaging

## Abstract

*Aloe Vera* is an ancient medicinal plant especially known for its beneficial properties for human health, due to its bioactive compounds. In this study, nanofibers with antioxidant activity were successfully obtained by electrospinning technique with the addition of a natural *Aloe Vera* skin extract (AVE) (at 0, 5, 10 and 20 wt% loadings) in poly(ethylene oxide) (PEO) solutions. The successful incorporation of AVE into PEO was evidenced by scanning electron microscopy (SEM), Fourier transform infrared spectroscopy (ATR-FTIR), thermogravimetric analysis (TGA) and antioxidant activity by 2,2-diphenyl-1-picrylhydrazyl radical scavenging (DPPH), 2,2′-azinobis-(3-ethylbenzothiazoline-6-sulfonic acid) radical scavenging (ABTS) and ferric reducing power (FRAP) assays. The incorporation of AVE introduced some changes in the PEO/AVE nanofibers morphology showing bimodal diameter distributions for AVE contents in the range 10-20 wt%. Some decrease in thermal stability with AVE addition, in terms of decomposition onset temperature, was also observed and it was more evident at high loading AVE contents (10 and 20 wt%). High encapsulation efficiencies of 92%, 76% and 105% according to DPPH, FRAP and ABTS assays, respectively, were obtained at 5 wt% AVE content, retaining AVE its antioxidant capacity in the PEO/AVE electrospun nanofibers. The results suggested that the obtained nanofibers could be promising materials for their application in active food packaging to decrease oxidation of packaged food during storage.

## 1. Introduction

The use of biopolymers or active biomolecules, obtained from agricultural by-products or wastes as a renewable source of materials to generate innovative added-value products, has gained great importance in the past few years, due to its environmental and economic advantages [1,2,3,4]. According to Tuck et al. [5], 2 × 10^11^ tons of lignocellulosic biomass residues are annually generated worldwide, being considered an unavoidable source of potential resources [6]. Several agrowastes such as *Aloe Vera* peel [7,8], almond skin [9], quercus bark [10], carob pods [11], tomato seeds [12] and coffee grounds [13] have been reported to contain biomolecules with interesting antioxidant/antimicrobial properties. These active compounds, once extracted from the vegetal matrices, could be potentially applied in the development of innovative materials for food packaging or edible coatings; as functional food ingredients, food additives or flavourings [14,15,16,17]; nutraceuticals and cosmetics, among other sectors [3].

*Aloe Vera* or *Aloe Barbadensis Miller* is an ancient medicinal plant, commonly associated with curative or healing properties, specially related to skin burns, wound or infections [18]. Some studies have also reported that *Aloe Vera* leaf possesses numerous activities including anticancer, antioxidant, antimicrobial, anti-inflammatory, antidiabetic and immunomodulatory [19,20,21]. *Aloe Vera* has become a popular ingredient in innumerable food, cosmetic and pharmaceutical products [18,22,23], using the colourless inner leaf gel in their formulation, which is first separated from the external skin, and then usually discarded generating big amounts of wastes. The *Aloe Vera* peel has not been fully investigated compared to the inner gel, but some studies have reported that this tissue could be an interesting source of active compounds with antioxidant and/or antimicrobial activity. This could be potentially be used as food additive, in active food packaging formulations or in the biomedical field for wound dressing or tissue engineering uses [24,25].

Conventional extraction techniques have been widely used to extract biomolecules from vegetal matrices. However, they present major drawbacks such as time consuming and the use of high quantities of solvents [26,27,28]. In recent years, alternative and more environmentally friendly extraction techniques have been developed. Among them, microwave-assisted extraction (MAE) has gained major importance due to its multiple advantages compared to conventional extraction techniques, leading to increased extraction yields and reduced extraction time and solvent consumption. MAE has been extensively used in the extraction of polyphenols from different natural sources [29,30,31,32].

Natural extracts rich in polyphenols, such as from *Aloe Vera,* usually show some instability under a wide range of processing or storage conditions being one of their major drawbacks for their final use. Polyphenols are particularly sensitive towards high temperature or humidity, light exposure, certain pH values and oxidation [33]. In this context, encapsulation techniques can provide the necessary protection of sensitive bioactive compounds against oxidation and decomposition for natural extracts [34]. Among the different available techniques, nanoencapsulation technologies such as nanoemulsification, electrospraying, electrospinning, production of nanoliposomes and solid lipid nanoparticles, nanostructures formation by cyclodextrins, etc., are very promising to entrap bioactive compounds [35]. Electrohydrodynamic processes, e.g., electrospinning and electrospraying, have gained increasing attention over the last decades [36,37,38] to obtain micron, submicron and nanometric fibres or nanoparticles, respectively, from a variety of materials, including synthetic and natural-based polymers. These techniques can be operated at room temperature and atmospheric pressure. They are particularly suitable for stabilization, protection, and controlled/targeted release of nanoencapsulated bioactive compounds [35], and avoid potentially detrimental effects that might occur when other techniques are applied [33,39].

Electrospun fibres have been used in a wide range of applications [40], such as in the biomedical field, including tissue engineering [41], wound dressing [42], drug delivery [43], and biosensors [44]; materials for catalysis and filtration [45]; and biomaterials for food packaging [37,46,47,48,49]. Several parameters could affect the fibres production process by electrospinning technique, which have to be rigorously controlled to obtain materials with desired characteristics [50,51]: Processing (applied voltage, distance between the tip of the needle and the collector and flow rate), solution (solvent, concentration, viscosity, conductivity) and environmental (temperature, relative humidity) parameters. A wide range of natural extracts with antioxidant activity, such as rosemary extract [52,53], grape seed extract [54,55,56], *Momordica charantia* fruit extract [57], medicinal plant extracts [58], pomegranate peel extract [59], yerba mate extract [60], garlic extract [42] and liquorice extract [43], were encapsulated in electrospun fibres. In addition, *Aloe Vera* inner gel extract was recently incorporated in different polymer matrices by electrospinning technique for several applications, mainly related to wound healing and skin tissue engineering, due to its intrinsic healing properties. In this sense, poly(vinyl alcohol) (PVA) [61], poly(ε-caprolactone) (PCL) [62,63,64], poly(vinylpyrrolidone) (PVP) [65], poly(lactic-co-glycolide acid) (PLGA) [66] and chitosan/PEO-based nanofibrous materials [67] including *Aloe Vera* gel with interesting functionalities (i.e., antioxidant, antimicrobial, enhanced mechanical properties) have been proposed for wound dressing applications. In relation to food packaging applications, *Aloe Vera* leaf juice was directly used as water-based solvent for PVP and PVA nanofibers production by electrospinning techniques [68].

Among the several electrospinnable polymers, PEO is a hydrophilic polymer that has been safely used in the medicine and food fields for the encapsulation of bioactive compounds by electrospinning, due to its non-toxicity, biocompatibility and biodegradability properties [33,69]. In addition, PEO has been approved by the Food and Drug Administration (FDA) as a safe food contact material [70]. PEO can be used to encapsulate a wide variety of antioxidant or antimicrobial compounds to obtain suitable nanofibers for active packaging applications. Different approaches have been reported for using PEO-based nanofibers in food packaging applications where the choice of an adequate foodstuff and package development (i.e., nanofibers, films, composites, single layer or multilayer, etc.), according to the limitations of the packaging material, are key aspects to be considered. Aydogdu et al. [71] enhanced the oxidative stability of walnuts by using gallic acid loaded into lentil flour/PEO nanofibers as active packaging materials. In other studies, antibacterial nisin-loaded nanoparticles embedded within PEO nanofibers as cheese packaging to improve the anti-*Listeria monocytogenes* activity [72] and plasma treated PEO electrospun mats containing tea tree essential oil/β-cyclodextrin inclusion complex to extend the beef shelf life [73] were developed. However, to the best of our knowledge, PEO nanofibers containing *Aloe Vera* skin extract have not been proposed and investigated yet, and no attention has been given to the study and application of electrospinning for the encapsulation of *Aloe Vera* skin extract (AVE), which has been considered the richest plant fraction with antioxidant compounds [8].

In this context, the main aim of the present study is the development of electrospun nanofibers with antioxidant activity by using poly(ethylene oxide) as matrix and different amounts (0, 5, 10 and 20 wt%) of an *Aloe Vera* skin extract, previously obtained by microwave-assisted extraction (MAE), as antioxidant agent. The obtained nanofibers were characterized in terms of morphology, structural properties, encapsulation efficiency, thermal stability and antioxidant activity.

## 2. Materials and Methods

### 2.1. Materials

Fresh *Aloe Vera* leaves from three-year old plants having a weight around 700–1000 g and a length of 50–70 cm were supplied by Las Coronas (Carnota, Sevilla, Spain). The tip, base and spikes of the leaves were removed and the epidermis was carefully separated from the inner gel using a sharp knife. The resulting *Aloe Vera* skin was intensively washed with distilled water and cut into small pieces. Then, it was freeze-dried with a Telstar Lyoquest −55 PLUS (Terrassa, Barcelona, Spain) and ground using a ZM 200 high-speed rotatory mill (Restch, Hann, Germany). Particles passing through a 1.0 mm sieve were used to ensure the homogeneity of the sample.

Poly(ethylene oxide) (PEO, Mw ≈ 500,000 Da), absolute ethanol (99.8%), sodium acetate, 2,2-diphenyl-1-picrylhydrazyl (DPPH), glacial acetic acid, hydrochloric acid, 2,4,6-tripyridyl-s-triazine (TPTZ), ferric chloride hexahydrate, potassium persulphate, 2,2′-azinobis-(3-ethylbenzothiazoline-6-sulfonic acid) diammonium salt (ABTS) and 6-hydroxy-2,5,7,8-tetramethylchroman-2-carboxylic acid (Trolox) were purchased from Sigma Aldrich (Madrid, Spain). Distilled-deionized water from a Millipore Milli-Q ultrapure water system was used (18.2 MΩ·cm at 25 °C).

### 2.2. Preparation of Aloe Vera Extract

Microwave-assisted extraction method was performed to obtain *Aloe Vera* extract (AVE) by following a procedure previously optimized. A Milestone Flexiwave (Milestone srl, Sorisole, Italy) was used in open vessel mode. 1.5 g of freeze-dried *Aloe Vera* skin powder were mixed with 50 mL of an ethanolic solution (80%, *v*/*v*) in a round-bottom flask. Then, the sample was heated in the microwave oven at 40 °C for 20 min under magnetic stirring (400 rpm). After the extraction, the supernatant was collected and stored overnight at −20 °C to remove possible interferences by precipitation. Subsequently, the supernatant was separated by centrifugation and ethanol was evaporated under reduced pressure. Finally, the *Aloe Vera* extract (AVE) was freeze-dried and stored at −20 °C in darkness until further use.

### 2.3. Preparation of Electrospun Nanofibers

PEO (5 wt%) was dissolved in milli-Q water under magnetic stirring for 24 h. PEO/AVE mixtures were prepared in order to obtain nanofibers with antioxidant activity by electrospinning. PEO and AVE concentrations were selected based on preliminary experiments considering the morphology of obtained nanofibers. AVE was previously dissolved in an ethanolic solution (80%, *v*/*v*) and it was added to PEO solution at different concentrations (0, 5, 10, and 20 wt% of AVE, with respect to the polymer content). All solutions were stirred in closed vials for 4 h in the dark to avoid any detrimental light effect.

The electrospinning process was carried out at room temperature with a homemade apparatus composed of a digitally controlled KDS-100-CE syringe pump (KD Scientific Inc, Holliston, MA, USA), a high voltage power supply (Spellman, SLM50P300, Hauppauge, NY, USA), a circular aluminium fixed collector and a 10.0 mL glass syringe. Different combinations of applied voltage, flow rate and distance between the needle (20 G, i.d. = 0.6 mm) and the collector were tested to select the optimal process parameters. Final optimized conditions were 16 kV, 0.5 mL h^−1^, and 15 cm, respectively. Experiments were performed at 25 ± 2 °C and 40–50% relative humidity. After the electrospinning process, the obtained mats were carefully separated from the collector and conveniently stored until further analysis.

### 2.4. Characterization of Electrospun Nanofibers

#### 2.4.1. Scanning Electron Microscopy (SEM)

SEM (Zeiss Leo Supra 35, Cambridge, UK) was employed to examine the morphology of PEO/AVE nanofibers. The fibres were mounted on aluminium stubs and then coated with an Au layer (≈5 nm) by sputtering (argon atmosphere, 25 mA, 7 × 10^−4^ Bar, 120 s) prior to SEM analysis. The average fibre diameter (AFD) of each fibrous mat was calculated by means of ImageJ software, considering at least 100 random points from the SEM images at 30k× magnification level. The results were reported as mean ± standard deviation.

#### 2.4.2. Fourier Transform Infrared Spectroscopy (FTIR)

FTIR spectra of pure components and PEO/AVE nanofibers were recorded using an infrared spectrophotometer in ATR mode (ATR-FTIR-4600 Jasco, Oklahoma City, OK, USA) in the 4000–600 cm^−1^ range (spectral resolution 4 cm^−1^, 32 scans).

#### 2.4.3. Thermal Analysis

Thermal properties of PEO/AVE electrospun nanofibers were evaluated by thermogravimetric analysis (TGA). TGA was carried out using a Mettler Toledo TGA/SDTA 851e equipment (Schwarzenbach, Switzerland). Approximately 5 mg of each sample were heated from room temperature up to 800 °C at 10 °C min^−1^ under nitrogen atmosphere (50 mL min^−1^). The onset temperature at 1% weight loss, temperature of maximum degradation and amount of residue at 800 °C were determined. Analyses were performed in triplicate.

#### 2.4.4. Antioxidant Activity

Antioxidant activity (AO) of PEO/AVE electrospun nanofibers was determined, in triplicate, by using three different methods: DPPH (2,2-diphenyl-1-picrylhydrazyl), FRAP (ferric reducing antioxidant power) and ABTS (2,2′-azinobis-(3-ethylbenzothiazoline-6-sulfonic acid) diammonium salt) assays.

##### DPPH Radical Scavenging Method

The DPPH scavenging activity was determined according to the method proposed by Aytac et al. [74] with some modifications, dissolving 1 mg of each PEO/AVE nanofiber sample in 2 mL of 10^−4^ mol L^−1^ freshly prepared DPPH ethanolic solution (80%, *v*/*v*). The absorbance was measured at 517 nm during 5 h using a Biomate-3 UV/Vis spectrophotometer (Thermospectronic, Mobile, AL, USA). The scavenging activity (%) was calculated as percentage of inhibition following Equation (1), where A_control_ and A_sample_ represent the absorbance values of the DPPH solution with, and without, the presence of the sample, respectively. 0.1 mL of AVE ethanolic solutions (80%, *v*/*v*) at different concentrations were mixed with 2 mL of the freshly prepared DPPH solution and the absorbance was measured following the same procedure. The inhibition of DPPH free radicals was also compared with a Trolox calibration curve (10–250 mg kg^−1^; 6 points, R^2^ = 0.9991), expressing results as µmol of Trolox equivalents per mg of nanofiber:

(1) $$\text{AO activity }\left(\%\right)=100\;\times \;\frac{A_{\mathrm{control}}-A_{\mathrm{sample}}}{A_{\mathrm{control}}}.$$

##### FRAP Method

The FRAP assay was determined according to Benzie and Strain [75]. The FRAP reagent was prepared mixing 0.3 mol L^−1^ acetate buffer (pH = 3.6), 10 mmol L^−1^ TPTZ made up in 40 mmol L^−1^ HCl and 20 mmol L^−1^ of aqueous FeCl_3_ at a 10:1:1 (*v*/*v*/*v*) ratio. Then, 1 mg of the PEO/AVE nanofiber samples was dissolved in 3 mL of the freshly prepared FRAP reagent pre-heated at 37 °C. The absorbance was measured at 593 nm during 5 h. 0.1 mL of AVE ethanolic solutions (80%, *v*/*v*) at different concentrations were also mixed with 3 mL of the FRAP working solution and the absorbance was measured analogously. Trolox was used as standard for preparing the calibration curve (10–250 mg kg^−1^; 6 points, R^2^ = 0.9988) and results were expressed as µmol of Trolox equivalents per mg of nanofiber.

##### ABTS Radical Scavenging Method

The ABTS assay was performed according to Quispe et al. [25]. The ABTS radical cation was produced by mixing the ABTS solution (7 mM) with 2.45 mM potassium persulfate in a 1:1 ratio and allowing the mixture to stand in the dark at room temperature for 12 h. The ABTS working solution was obtained by diluting with aqueous ethanol (80%, *v*/*v*) to a final absorbance of 1.00 ± 0.01 at 734 nm. 1 mg of the PEO/AVE nanofiber samples was dissolved in 3 mL of the freshly prepared working ABTS solution and the absorbance was measured during 5 h. 0.1 mL of AVE ethanolic solutions (80%, *v*/*v*) at different concentrations were also mixed with 3 mL of the ABTS working solution and the absorbance was measured following the same procedure. The AO activity (%) was calculated analogously to the DPPH method using Equation (1). The inhibition of ABTS radicals was also compared with a Trolox calibration curve (10–250 mg kg^−1^; 6 points, R^2^ = 0.9995), expressing results as µmol of Trolox equivalents per mg of nanofiber.

#### 2.4.5. Encapsulation Efficiency

The encapsulation efficiency (EE) of AVE in the mat of fibres obtained after the electrospinning process was calculated according to Equation (2):

(2) $$\mathrm{EE}\;\left(\%\right)=100\;\times \;\frac{\text{amount of AVE calculated from AO assay}}{\text{initial amount of AVE used in the polymer formulation}}$$

considering the AVE content determined by the antioxidant assays and the AVE amount used in the polymer formulations. The measurements were performed in triplicate.

### 2.5. Statistical Analysis

Statistical analysis of results was performed with Statgraphics Centurion XVI statistical software. An analysis of variance (ANOVA) was carried out. Differences between average values were assessed based on the Tukey test at a confidence level of 95% (*p* < 0.05).

## 3. Results

Poly(ethylene oxide) (PEO) is a water-soluble, non-toxic, biocompatible and biodegradable polymer that can produce bead-free fibres under a wide combination of process parameters, alone or combined with other polymers [76,77,78,79,80]. The encapsulation of AVE in the electrospun nanofibers was confirmed by SEM, FTIR, TGA and antioxidant assays. In addition, the AVE incorporation was demonstrated by showing the obtained electrospun nanofibers a characteristic brownish colour and easily AVE recognizable fragrance.

### 3.1. SEM

**Figure 1:**
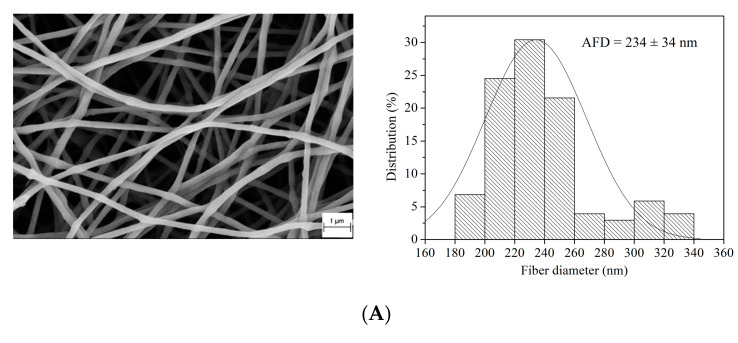

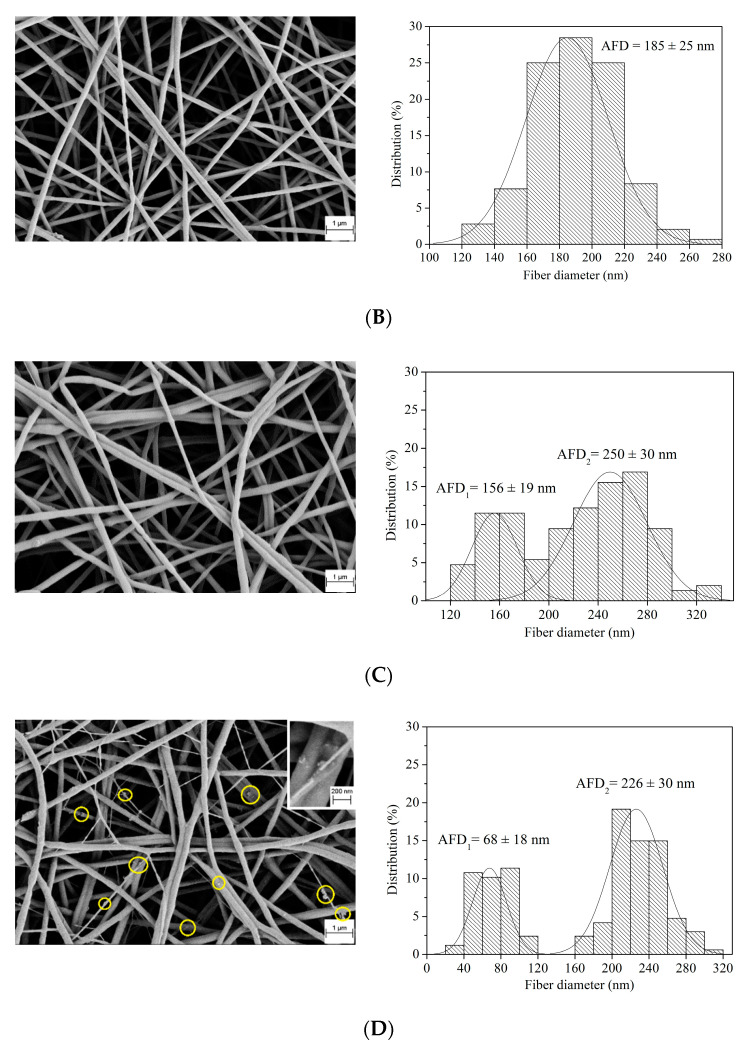
SEM micrographs and diameter distribution of PEO/AVE nanofibers. (**A**) PEO; (**B**) PEO/AVE 5 wt%; (**C**) PEO/AVE 10 wt%; (**D**) PEO/AVE 20 wt%. Yellow circles in Figure 1D indicate AVE particles.

The morphology and size of PEO/AVE nanofibers were studied by SEM. Figure 1 shows the SEM micrographs, size distribution and average fibre diameter (AFD) of the electrospun nanofibers. Smooth and bead-free non-woven and randomly oriented nanofibers were successfully obtained for all formulations. The presence of AVE in the nanofibers was confirmed by the observation of little particles homogeneously distributed throughout the non-woven mat of fibres, especially at higher AVE content (20 wt%) (Figure 1D). Neat PEO and low AVE content PEO/AVE nanofibers exhibited typical symmetric normal diameter distributions with one clearly defined AFD. 234 ± 34 nm and 185 ± 25 nm were obtained for neat PEO, and PEO with 5 wt% AVE, respectively. This decrease in fibre diameter with the addition of AVE could be related to a decrease in viscosity and/or higher electrical conductivity of the solutions [71,81,82]. An increase in the AVE content induced a different behaviour in the electrospun nanofibers production, as it is shown in Figure 1C,D, observing bimodal diameter distributions with two completely differentiated AFDs. The addition of AVE at 20 wt% to PEO produced the formation of a first group of thinner nanofibers compared to PEO/AVE at 10 wt%. This nanofiber inhomogeneity could be a qualitative indicator of AVE encapsulation in the PEO fibres as a consequence of the mixing of the two different solutions resulting in heterogeneous fibre diameters at high AVE loadings. Therefore, the addition of AVE could introduce some changes in viscosity, rheological properties and/or conductivity of polymer solutions, which may play a critical role in the control of the morphology and size of the obtained electrospun nanofibers [83,84,85].

### 3.2. FTIR

**Figure 2:**
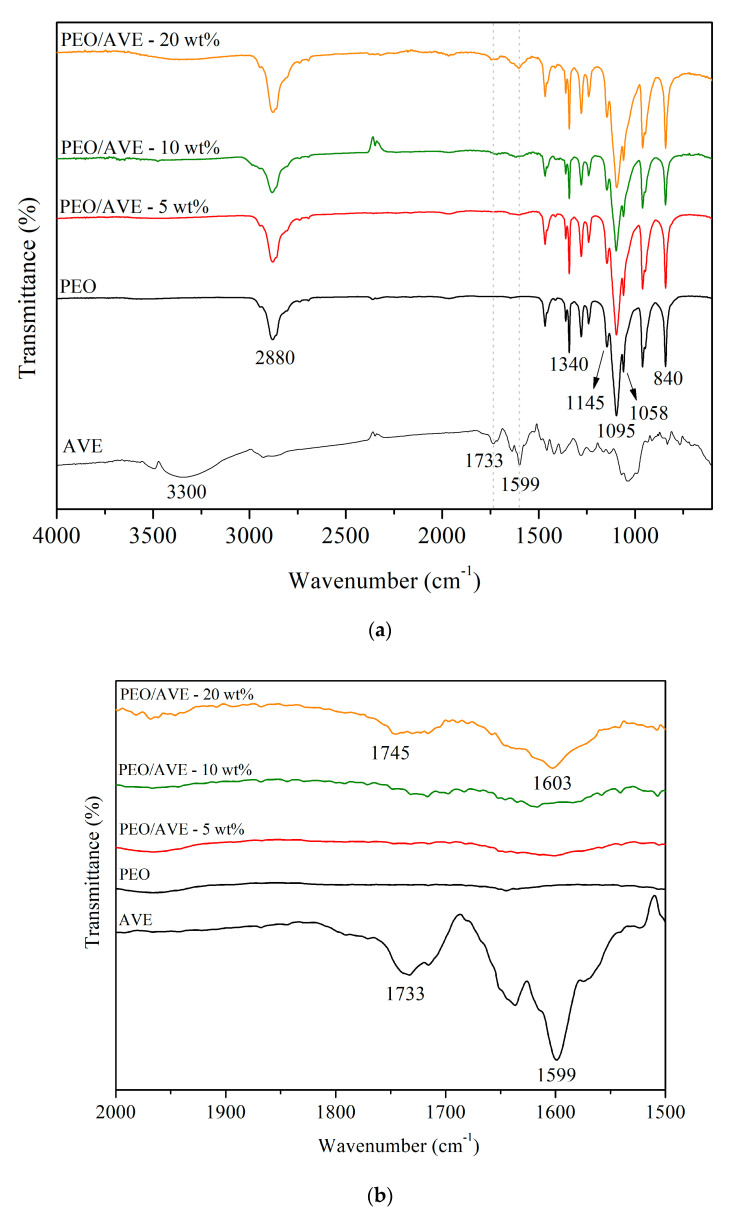
FTIR spectra of AVE, and PEO and PEO/AVE nanofibers. (**a**) from 4000 to 600 cm^−1^ (**b**) zoom region from 2000 to 1500 cm^−1^.

The ATR-FTIR spectra of AVE and PEO and PEO/AVE nanofibers are shown in Figure 2. AVE is a complex mixture of bioactive compounds that are naturally present in *Aloe Vera* and it might include different aromatic and phenolic groups (flavonoids, cinnamic acids and derivatives, chromones, anthracene compounds, among others) [24,25]. The FTIR spectrum of AVE displayed the characteristic broad band of phenolic compounds at 3330 cm^−1^ corresponding to stretching modes of the different −OH groups. Furthermore, the peaks at 1733 and 1599 cm^−1^ could be attributed to C = O, and C = C ring stretching, respectively [54].

The FTIR spectrum of PEO nanofibers (Figure 2) showed two peaks at 2880 and 1340 cm^−1^ which could be assigned to C–H stretching, and CH_2_ wagging vibration, respectively. The characteristic triplet peak at 1145, 1095, and 1058 cm^−1^ was correlated to the asymmetric and symmetric stretching of C–O–C. Moreover, the band at 840 cm^−1^ was attributed to the stretching vibration of the molecular bond C–O–C and the C–C. These results were in close agreement with those observed in previous works [58,71,76,83,86,87]. The FTIR spectra of PEO/AVE nanofibers showed the characteristic AVE absorbance peaks at 1745 and 1603 cm^−1^ (Figure 2b), with higher intensity with increasing AVE content in the polymer formulations used for nanofibers production. These bands were shifted with respect to those observed for pure AVE, suggesting an interaction between the polymer matrix and AVE, probably based on intermolecular hydrogen bonds [54]. These results confirmed the successful incorporation of AVE into the PEO mats of fibres after the electrospinning process. Authors have also demonstrated the presence of active compounds from natural extracts in electrospun nanofibers through the identification of characteristic peaks in FTIR spectra. For instance, the peaks observed around 1600 cm^−1^ for grape seed [54,56], rosemary [52] and *Garcinia mangostana* [88] extracts were used to verify the incorporation of these active additives in the nanofibers after electrospinning.

### 3.3. Thermogravimetric Analysis

**Figure 3:**
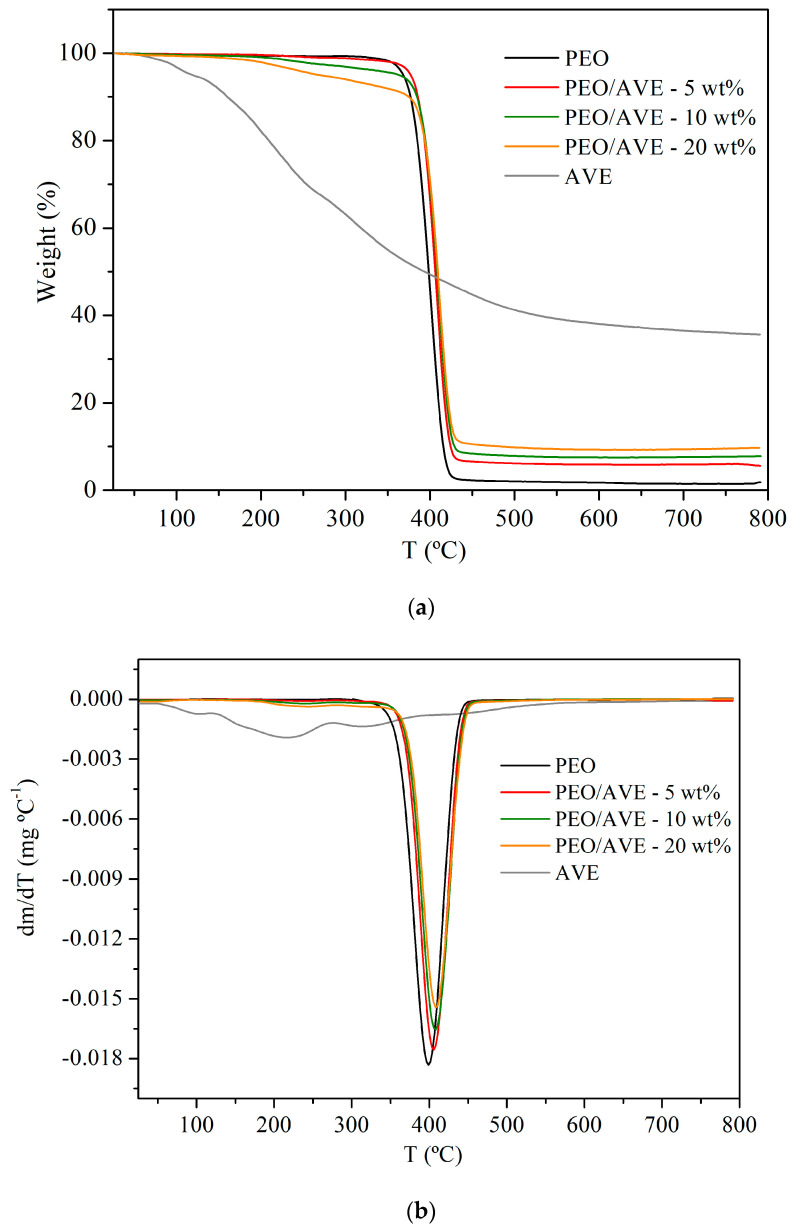
(**a**) TGA and (**b**) DTG thermograms of AVE, and PEO and PEO/AVE nanofibers.

TGA and derivative (DTG) curves of AVE and PEO and PEO/AVE nanofibers are presented in Figure 3 and they display the weight loss from room temperature to 800 °C. For AVE, an initial step of mass loss was observed below 100 °C which was associated to water desorption [40]. This step was followed by a degradation process, which took place in an extended temperature range, suggesting that many overlapping processes could occur as a consequence of the complex composition of AVE, which is composed of different biomolecules present in the *Aloe Vera* skin [25,89,90]. This degradation comprised approximately 60% of the initial weight. The shown behaviour is in agreement with other authors who reported similar wide thermal degradation processes for other natural extracts such as rosemary extract [52], *Chimonanthus praecox* extract [91], and grape seeds extract [54].

PEO nanofibers degraded in one single step until approximately 400 °C with a decomposition onset temperature of 339 °C, and comprising nearly 98% of the initial weight (Table 1), in agreement with reported results by other authors [48,53,56]. PEO/AVE nanofibers also decomposed in a single-stage event (Figure 3). A decremented thermal stability was observed for PEO/AVE nanofibers, decreasing the decomposition onset temperature T_1%_ with increasing AVE content in the polymer formulations. This behaviour was more evident at high AVE loading, showing a decrease in T_1%_ of approximately 21, 40 and 55% for 5, 10 and 20 wt%, respectively, which was associated with the initial thermal degradation of AVE present in the polymer formulations. Locilento et al. found a similar behaviour with decreasing thermal stability of the resulted electrospun nanofibers upon encapsulation of grape seeds extract, which was attributed to the dissolution/dispersion of the bioactive compound before encapsulation [54]. However, a minor increase in the temperature of maximum degradation of PEO/AVE nanofibers was observed with the addition of AVE, showing no significant differences occurred (*p* > 0.05) with the increase in AVE content (Table 1). This result suggests that although the incorporation of AVE seems to cause some initial decrease in stability, it does not affect the overall maximum decomposition of the developed electrospun PEO/AVE nanofibers. The amount of residue remaining at the end of the degradation process significantly increased (*p* < 0.05) with the AVE content in the nanofibers (Table 1). As a result, the inclusion of AVE in the final electrospun nanofibers was demonstrated by TGA results although the amount of AVE remaining in each formulation after the electrospinning process could not be accurately estimated due to the overlapping of the degradation processes of neat PEO and AVE (Figure 3).

### 3.4. Antioxidant Activity and Encapsulation Efficiency

**Figure 4:**
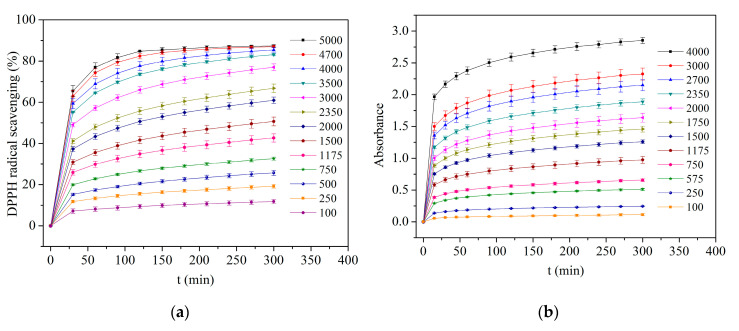

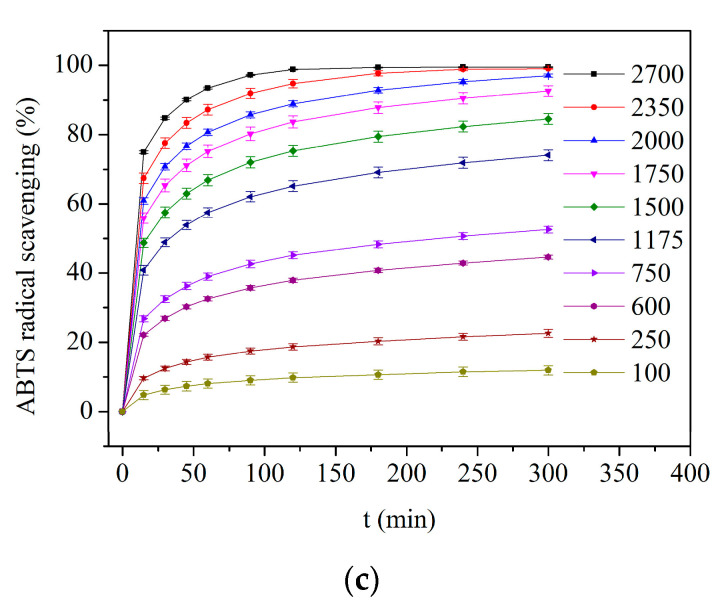
Antioxidant activity of AVE solutions at different concentration levels with time by (**a**) DPPH (**b**) FRAP and (**c**) ABTS assays. AVE concentration levels are expressed in mg_AVE_ kg^−1^.

The antioxidant properties of *Aloe Vera* extracts have been linked to a wide variety of compounds showing different reducing and radical quenching abilities, including hydroxycinnamic acid derivatives such as chlorogenic, caffeic, ferulic and sinapic acid; chromones (1,4-benzopyrone derivatives); anthrones (10H-anthracen-9-one derivatives); and flavonoids, such as catechin, quercetin, myricetin and luteolin [8,24,25,92]. Moreover, *Aloe Vera* leaf skin has been reported to be the most active fraction of the plant and more than twenty-five active compounds have been identified in extracts from different parts of the plant including peel, gel, flowers and roots [8,92]. The antioxidant activity of flavonoids, phenolic acids and their derivatives depends on their chemical structures, since it is strongly influenced by the number and position of the hydroxyl groups and the presence of certain moieties, such as catechol in the aromatic rings or conjugated double bonds with carbonyl groups in the C-ring [93]. In addition, it has been reported that the ability of polyphenolic compounds to act as antioxidants depends on the redox properties of their phenolic hydroxyl groups and their potential for electron delocalization across the chemical structure [94]. Since natural plant extracts such as AVE are composed of several bioactive compounds with different antioxidant activity exerted through different action mechanisms and possible synergistic interactions, it is necessary to combine different methods to determine the in vitro antioxidant capacity [95]. Among them, DPPH, FRAP and ABTS methods have been widely used to evaluate the antioxidant activity of many pure compounds and natural extracts [96,97,98]. In addition, these methods have been extensively applied to study the antioxidant activity of different types of materials such as fibres produced by electrospinning [99,100,101,102], films for active packaging systems [103,104,105], and nanoparticles [106,107]. These methods were used in the present study to determine the antioxidant activity of the PEO/AVE nanofibers. Figure 4 shows the antioxidant activity of AVE solutions prepared at different concentration levels (mg_AVE_ kg^−1^) as a function of time by all the studied AO assays. As it can be observed, an increasing trend in antioxidant activity with increasing AVE concentration was observed until reaching a steady-state at approximately 300 min in all cases. These results proved the direct correlation of the AO assays used to study the antioxidant activity of AVE solutions.

**Figure 5:**
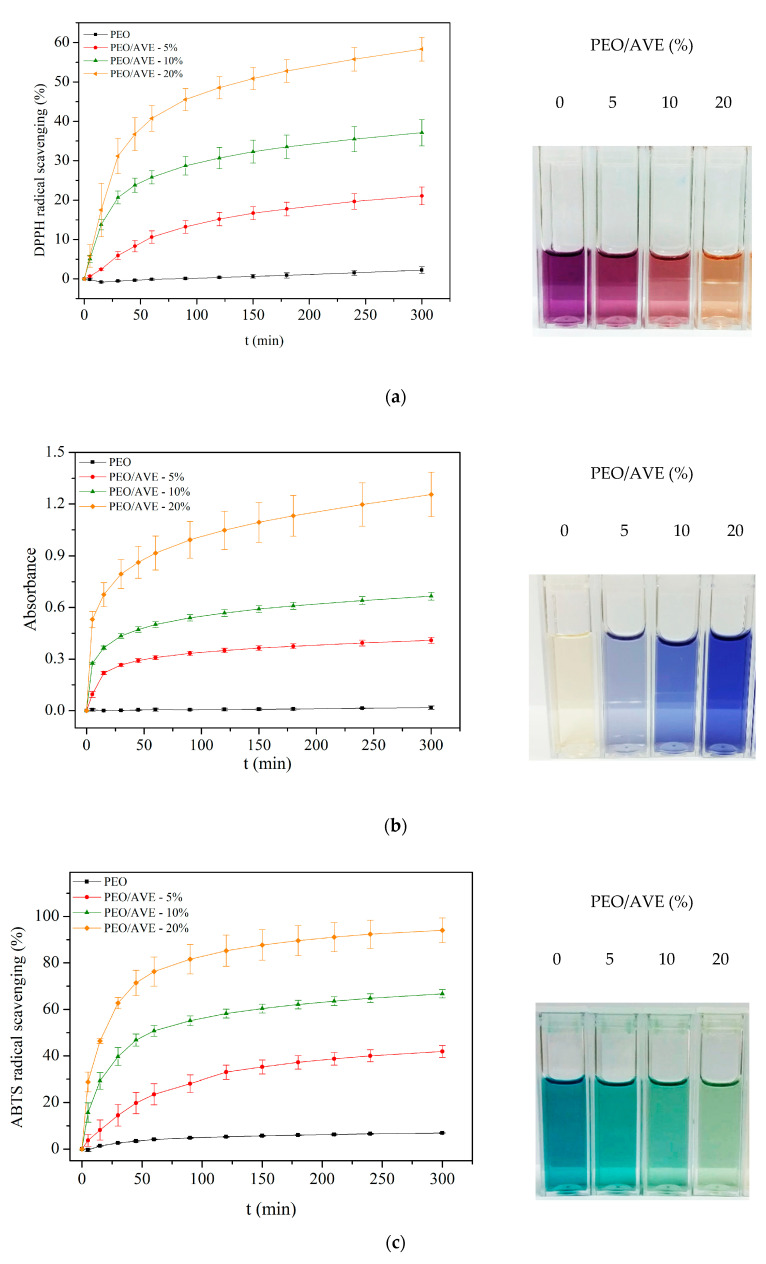
Antioxidant activity of PEO/AVE nanofibers with time by (**a**) DPPH (**b**) FRAP and (**c**) ABTS assays. Final colour solutions are shown at t = 300 min.

The antioxidant activity of PEO/AVE nanofibers with time for all AO assays is displayed in Figure 5. As expected from Figure 4, an increasing trend in antioxidant activity with increasing AVE amount in the PEO/AVE nanofibers was revealed, observing clearly colour changes, depending on the AVE content present in the nanofibers. The encapsulation efficiency (EE) of AVE in the electrospun nanofibers was higher at 5 wt% loading, as shown in Table 2, obtaining 92%, 76% and 105% according to DPPH, FRAP and ABTS assays, respectively. For higher AVE contents, the EE slightly decreased with increasing the initial AVE loading in the polymer solutions prior to electrospinning, although relatively high encapsulation efficiencies were obtained in all cases. Some minor decrease was specially observed with the ABTS assay for 20 wt% AVE loading compared to the other PEO/AVE nanofibers. These differences in EE could be attributed to the different reaction mechanisms of the several methods used, in particular from FRAP to DPPH and ABTS assays. DPPH and ABTS radical scavenging assays are based on the reduction of DPPH or ABTS molecule by a hydrogen donor antioxidant and the colour of the solutions change from purple to yellow, and from green to yellow, respectively [108,109]. On the other hand, the FRAP assay measures the ferric reducing ability of plasma at low pH when a ferric-tripyridyltriazine complex was reduced to its ferrous form, developing an intense blue colour [75]. In this sense, the applied antioxidant assays may evaluate different fractions of antioxidant species that might partially overlap each other. It has to be considered that the concentrations of individual antioxidants in AVE are not the only factor influencing antioxidant capacity; indeed, the structural arrangements (number and position of hydroxyl groups, double bonds, and aromatic rings) of these compounds also play an important role [110]. Therefore, their individual contributions to DPPH, ABTS and FRAP may also differ. While the DPPH and ABTS methods evaluate the free radical scavenging capacity of a sample, the FRAP method evaluates the content of electron-donating species with a certain redox potential [96]. As a consequence, the different individual components present in AVE may have stronger free radical scavenging abilities than reducing power, or vice versa, dependent on their chemical structures, giving different results as shown in Table 2. It should be also mentioned that the DPPH method may not be sensitive to some antioxidant molecules, in particular, to those of a larger size [93]. Consequently, the results of the DPPH assay could underestimate the radical absorbance capacity of some extracts to a degree [96]. Finally, FRAP activity has been reported to be also dependent on the steric hindrance between antioxidants and ferric di-TPTZ complex [111,112].

Total AO activity of AVE loaded electrospun nanofibers at t = 300 min is shown in Table 2. In all cases, PEO/AVE nanofibers showed antioxidant activity evidencing the successful encapsulation of AVE by electrospinning. Moreover, the total antioxidant activity of nanofibers expressed as µmol_Trolox_ mg_fibre_^−1^ increased significantly (*p* < 0.05) with increasing AVE loading, being directly correlated to the AVE content found in the nanofibers. These findings are in agreement with other authors who observed similar relationships between AO activity of fibres and active compound contents found in the materials. For instance, Aydogdu et al. [113] reported increasing AO activity of hydroxypropyl methyl cellulose/PEO fibres with increasing gallic acid content in the polymer formulations. Similarly, Vatankhah [53] observed higher AO activity of cellulose acetate fibres when higher rosmarinic acid content was encapsulated. Furthermore, many authors demonstrated the antioxidant activity of polymer fibres or films incorporating natural extracts intended for food packaging applications [54,71,114,115,116] and similar antioxidant values were obtained by using different natural extracts. Estevez-Areco et al. [52] revealed an AO activity of 0.120 µmol_Trolox_ mg^−1^ for electrospun PVA fibres loaded with rosemary extract, Araújo et al. [117] 0.013 µmol_Trolox_ mg^−1^ for starch films loaded with an ethanolic propolis extract, De Freitas et al. [118] 0.005 µmol_Trolox_ mg^−1^ in zein films including pinhão extract, and Genskowsky et al. [119] 0.011 µmol_Trolox_ mg^−1^ in chitosan films loaded with maqui berry extract.

**Figure 6:**
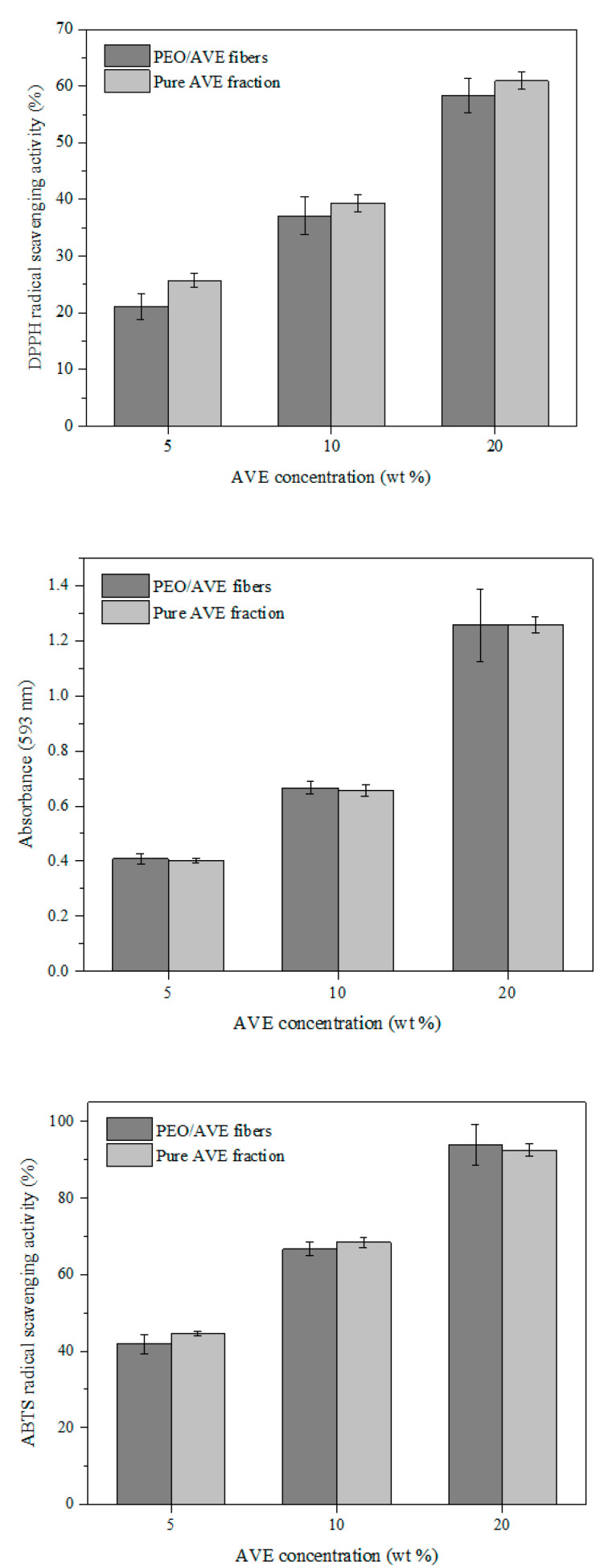
Antioxidant activity of PEO/AVE nanofibers and pure AVE fractions at t = 300 min. Error bars indicate standard deviation.

On the other hand, it has been reported that encapsulation techniques could enhance the AO activity of natural compounds such as carotenoids [111] and curcumin [112], compared to their non-encapsulated forms. Aceituno-Medina et al. [120] also found an improved AO capacity of quercetin and ferulic acid during in vitro digestion through encapsulation within food-grade electrospun fibers. This behavior might be related to a decrease in extent of aggregation of the encapsulated bioactive compounds, a greater specific surface of nanoparticles, allowing active molecules to be more accessible to react with free-radical scavengers at the interface and/or the reduced steric hindrance of active molecules after encapsulation. This favours the reaction with FRAP reagent [111,112]. Figure 6 shows a comparison between the AO activity of PEO/AVE fibres and pure AVE fractions. Similar AO values were obtained for DPPH, ABTS and FRAP assays, indicating that the AO activity of AVE was maintained in a very high extent after the electrospinning process, ensuring the chemical integrity of AVE. Analogous results were obtained by Amna et al. [121] for the encapsulation of capsaicin into polyurethane-based electrospun fibres. Sarkar et al. [122] found a decrease in AO activity of honey components, compared to the non-encapsulated active sample after the production of PVA/honey electrospun fiber membranes. Similarly, Hosseini et al. [123] observed differences in DPPH and FRAP results of antioxidant fish peptide before and after its encapsulation into chitosan/poly(vinyl alcohol) electrospun nanofibers. The observed AO decrease for nanoparticles compared to the pure peptide fraction was related to the applied process conditions and the interactions that took place between the active agent and polymer during the electrospinning process. In the case of FRAP, this behaviour was associated with a longer time needed for the entrapped biomolecule to be released into the FRAP solution for the reducing ability to take place.

Despite the high voltage applied during the electrospinning process, it can be concluded that AVE preserved its original bioactivity after its incorporation in the polymer mixtures. It was successfully incorporated within PEO fibres by electrospinning technique, allowing obtaining nanofibers with quite high antioxidant activity. In addition, the electrospinning process was operated at room temperature and this may contribute to avoid and/or limit thermal degradation processes for thermolabile compounds present in natural extracts [42,43], such as AVE. PEO nanofibers showed negligible antioxidant activity by DPPH, FRAP and ABTS assays, as expected.

## 4. Conclusions

In this study, smooth defect-free non-woven and self-standing electrospun nanofibers were obtained using electrospinning technology and PEO as polymer matrix. A natural extract from *Aloe Vera* skin was incorporated in the polymer formulations, in order to produce PEO/AVE nanofibers with antioxidant capacity. The obtained materials were characterized by SEM, TGA, FTIR and three different antioxidant activity assays (DPPH, FRAP and ABTS). The results demonstrated that AVE was successfully encapsulated in the electrospun nanofibers, showing high encapsulation efficiencies, without important AVE losses during the electrospinning process, and maintaining its intrinsic antioxidant activity. The antioxidant capacity of the nanofibers was directly correlated with the AVE content in the polymer mixture prior to electrospinning. As a result, the electrospinning process was an appropriate method for successfully encapsulating the thermolabile bioactive extracts, such as AVE, due to its operating conditions at room temperature. The obtained PEO/AVE nanofibers, in particular those added with 5 wt% AVE, could be considered promising biodegradable materials with potential application in the development of innovative food packaging systems to enhance the oxidative stability of foods or as antioxidant wound dressings in biomedical sciences. In addition, the incorporation of this waste extract rich in antioxidants into PEO electrospun nanofibers could be an interesting approach to revalorise *Aloe Vera* by-products, contributing to the circular economy.

## Figures and Tables

**Table 1:** Thermal decomposition parameters of PEO/AVE nanofibers.

Sample	T_1%_ (°C)	* T_MAX_ (°C)	* %RES_800 °C_
PEO	339.0	399.6 ± 1.0 ^a^	2.0 ± 0.8 ^a^
PEO/AVE 5%	268.3	405.9 ± 0.2 ^b^	5.9 ± 0.4 ^b^
PEO/AVE 10%	204.8	408.6 ± 0.0 ^c^	7.0 ± 0.9 ^b,c^
PEO/AVE 20%	153.8	408.5 ± 0.5 ^c^	9.8 ± 0.6 ^c^

T_1%_: onset temperature at 1% weight loss; T_MAX_: temperature of maximum degradation; %RES_800__°C_: amount of residue present at 800 °C. * Mean ± SD (n = 3). Different superscripts (a–c) within the same column indicate statistically different values (*p* < 0.05).

**Table 2:** Antioxidant activity of PEO/AVE nanofibers (t = 300 min). Mean ± SD (n = 3).

Assay	Sample	m_AVE,f_ (mg)	EE (%)	µmol_Trolox_/mg_fiber_
DPPH	PEO	-	-	-
	PEO/AVE 5%	0.046 ± 0.009 ^a^	92.0 ± 18.1 ^a^	0.019 ± 0.002 ^a^
	PEO/AVE 10%	0.087 ± 0.014 ^b^	87.3 ± 13.7 ^a^	0.029 ± 0.003 ^b^
	PEO/AVE 20%	0.174 ± 0.012 ^c^	87.2 ± 6.2 ^a^	0.051 ± 0.003 ^c^
FRAP	PEO	-	-	-
	PEO/AVE 5%	0.038 ± 0.002 ^a^	75.8 ± 4.0 ^a^	0.028 ± 0.001 ^a^
	PEO/AVE 10%	0.066 ± 0.002 ^b^	65.9 ± 2.5 ^a^	0.046 ± 0.002 ^b^
	PEO/AVE 20%	0.130 ± 0.014 ^c^	65.1 ± 7.1 ^a^	0.086 ± 0.009 ^c^
ABTS	PEO	-	-	-
	PEO/AVE 5%	0.053 ± 0.004 ^a^	105.2 ± 8.4 ^a^	0.039 ± 0.002 ^a^
	PEO/AVE 10%	0.094 ± 0.003 ^b^	93.8 ± 3.0 ^a^	0.062 ± 0.002 ^b^
	PEO/AVE 20%	0.139 ± 0.009 ^c^	69.5 ± 4.4 ^b^	0.087 ± 0.005 ^c^

m_AVE,f_: amount of AVE calculated from AO assays. EE: encapsulation efficiency. Different superscripts (a,b,c) within the same column and AO assay indicate statistically different values (*p* < 0.05).
